# Task-Parceling and Synchronous Retrieval Scheme for Twin-Arm Orchard Apple Tree Automaton

**DOI:** 10.3390/plants14172798

**Published:** 2025-09-06

**Authors:** Bin Yan, Xiameng Li

**Affiliations:** 1 College of Automation and Information Engineering, Xi’an University of Technology, Xi’an 710048, China; 2 College of Mechanical and Electronic Engineering, Northwest A&F University, Yangling 712100, China; 3 Faculty of Liberal Arts, Northwest University, Xi’an 710127, China

**Keywords:** sustainable agriculture, robots, sensor systems, camera-based solutions, apple tree

## Abstract

To address suboptimal throughput performance in conventional intelligent apple harvesting systems predominantly employing single manipulators, a dual-arm harvesting robot prototype was engineered. Leveraging the AUBO-i5 manipulator framework and kinematic characteristics, a coordinated workspace arrangement was established. Subsequently, the dual-manipulator harvesting platform was fabricated. A dynamic task allocation methodology and intelligent fruit sequencing approach were formulated, grounded in U-tube optimization principles. This framework achieved parallel operation ratios between 82.1% and 99%, with combined trajectory lengths spanning 9.24–11.90 m. Building upon established apple harvesting knowledge, a sequencing strategy incorporating dynamic manipulator zoning was developed. Validation was conducted through V-REP kinematic simulations where end-effector poses were continuously tracked, confirming zero limb interference during coordinated motion. Field assessments yielded parallel operation rates of 85.7–93.3%, total harvest durations of 17.8–22.3 s, and inter-manipulator path differentials of 267–541 mm. Throughout testing, collision-free operation was maintained while successfully harvesting all target fruits according to planned sequences. These outcomes validate the efficacy of U-tube-based dynamic zoning and sequencing methodologies for dual-manipulator fruit harvesting in intelligent orchard applications.

## 1. Introduction

Amidst accelerating global agricultural automation [1,2,3], the fruit production sector [4,5,6,7]—a critical agricultural segment—confronts intensifying contradictions between escalating production requirements and insufficient workforce availability. Conventional manual harvesting approaches are characterized by intensive labor demands and suboptimal efficiency, while being progressively exacerbated by demographic aging trends and elevated labor expenditures [8,9,10,11]. Consequently, development of efficient robotic harvesting systems is recognized as an essential pathway for addressing apple harvesting challenges and enabling sustainable sector advancement [12]. Real-world orchard harvesting presents multifaceted challenges beyond robotic coordination: fruit integrity preservation demands delicate force-controlled grasping and stem-severing to prevent bruising that compromises commercial value; occlusion-heavy identification in dense canopy environments requires robust perception systems to overcome lighting variations, leaf/branch obstructions, and fruit clustering; terrain adaptability necessitates stable navigation and precise positioning on uneven, muddy, or sloped ground while compensating for platform vibrations; environmental variability including wind-induced fruit/tree motion and weather extremes (humidity, dust) further complicates reliable operation.

A unimanual apple harvesting system has been engineered, incorporating a triple-fingered end-effector and ZED stereoscopic vision technology. This apparatus employs a 5-DOF manipulator to execute peduncle separation through gripping maneuvers. Orchard validation indicates 26-millisecond computational latency per image frame during visual processing, with individual harvesting operations averaging 12.5 s and achieving 81.6% efficacy [13]. Vacuum-based harvesting apparatus developed by Abundant Robotics [14] demonstrates 1.5 s fruit detachment cycles, though its configuration targets standardized international orchards utilizing trellised V-configuration planting systems. However, spindle-trained apple cultivars prevalent in domestic orchards present heightened robotic adaptation challenges due to dense canopy obstruction phenomena. Additionally, bimanual coordination methodology for kiwifruit harvesting has been established, where operational zone segmentation is modeled as a multiple traveling salesmen problem [15]. A bimanual apple harvesting robotic platform was likewise developed, implementing genetic algorithm-based multi-manipulator task optimization to enhance collaborative efficiency through harvesting sequence refinement [12]. In the above studies, some were focused on the picking planning of non-apple targets using dual robotic arms, such as kiwi fruit dual-arm picking robots. Their planning methods and characteristics are applicable to the orchard growth environment and plant physical characteristics of kiwifruit, and therefore are not suitable for intelligent picking of apple targets. In addition, there are related studies on apple double-arm picking robots, but due to the use of a Cartesian coordinate system-type robotic arm, its picking planning algorithm is not directly applicable to the six-degrees-of-freedom robotic arm motion used in this study. The U-shaped tube double-arm picking task allocation optimization algorithm proposed in this paper has its own characteristics.

Crucially, the dual-arm design aligns with the spatial constraints and fruit distribution patterns typical of spindle-shaped apple trees in Chinese orchards. The semi-overlapping workspace configuration, optimized via the U-shaped tube algorithm, maximizes coverage while minimizing interference, a balance that would be exponentially harder to achieve with three or more arms due to increased kinematic complexity, collision risks, and control challenges. Adding more arms would also escalate hardware costs, energy consumption, and system weight, potentially compromising the robot’s mobility and practicality in dense orchard environments. Thus, the dual-arm system represents the most effective solution to address the core deficiency of single-arm systems (low efficiency) while avoiding the impracticalities of over-engineering, thereby delivering a scalable, collision-safe, and economically viable platform for intelligent harvesting.

Dual-arm apple harvesting robots demonstrate significant potential for improving harvesting efficiency due to the advantages of cooperative operation between their two robotic arms. Compared to single-arm systems, they can more efficiently execute a series of operations such as fruit grasping [16], harvesting [17,18], and transport. However, to achieve efficient and precise operation for dual-arm robots, two core issues urgently need resolution: dynamic partitioning for dual-arm harvesting operations and intelligent planning of the fruit picking sequence.

Operation partitioning is fundamental for efficient dual-arm collaborative work. In real orchard environments, the lack of reasonable zone division will lead to movement interference between the robotic arms, causing harvesting tasks to fail. A scientific dual-arm partitioning strategy can divide the harvesting area into different subspaces based on the layout characteristics of the robotic arms, enabling the robot to operate within predefined zones and ensuring operational safety. Simultaneously, this partitioning framework provides a structured basis for subsequent picking sequence planning.

Intelligent planning of the fruit picking sequence is a critical factor determining the operational efficiency of the robot. Within the subareas for which each robotic arm is responsible, an improper picking sequence will trigger frequent posture adjustments, increasing movement distance and joint angle variations, thereby reducing harvesting speed and accuracy. Furthermore, poor picking sequence planning may cause damage to fruits or trees, affecting fruit quality and subsequent growth. Therefore, it is necessary to establish an intelligent fruit picking sequence planning method tailored to dual-arm harvesting partitions, ensuring the dual-arm system safely and reliably completes tasks.

Despite extensive scholarly investigations into intelligent agricultural equipment operational planning [19,20,21,22,23,24], research addressing adaptive zoning methodologies for bimanual apple harvesting systems in orchard environments remains underdeveloped, particularly regarding multifactor-integrated harvest sequencing approaches. Confronting limitations in prevalent unimanual apple harvesting systems requiring efficiency enhancements, a bimanual harvesting robotic prototype was constructed. An innovative methodology was introduced: pose-constrained adaptive task zoning employing a U-tube optimization protocol for resource distribution. Additionally, leveraging horticultural expertise, a harvest sequencing strategy oriented toward bimanual adaptive zoning was formulated.

These challenges are effectively mitigated through the proposed adaptive zoning and intelligent sequencing framework for bimanual harvesting systems. The investigation’s significance is manifested in three dimensions: substantial enhancements in automated apple harvesting efficiency are projected; operational expenditures are reduced; theoretical foundations and technical implementation frameworks are established for practical deployment of bimanual harvesting platforms. Consequently, advancements in intelligent harvesting theory and technical capabilities within the apple industry are facilitated. These contributions provide substantive technical support for agricultural practitioners and agri-robotics equipment manufacturers.

## 2. Materials and Methods

### 2.1. Spatial Layout of Intelligent Picking Robot with Double Manipulator

**Figure 1:**
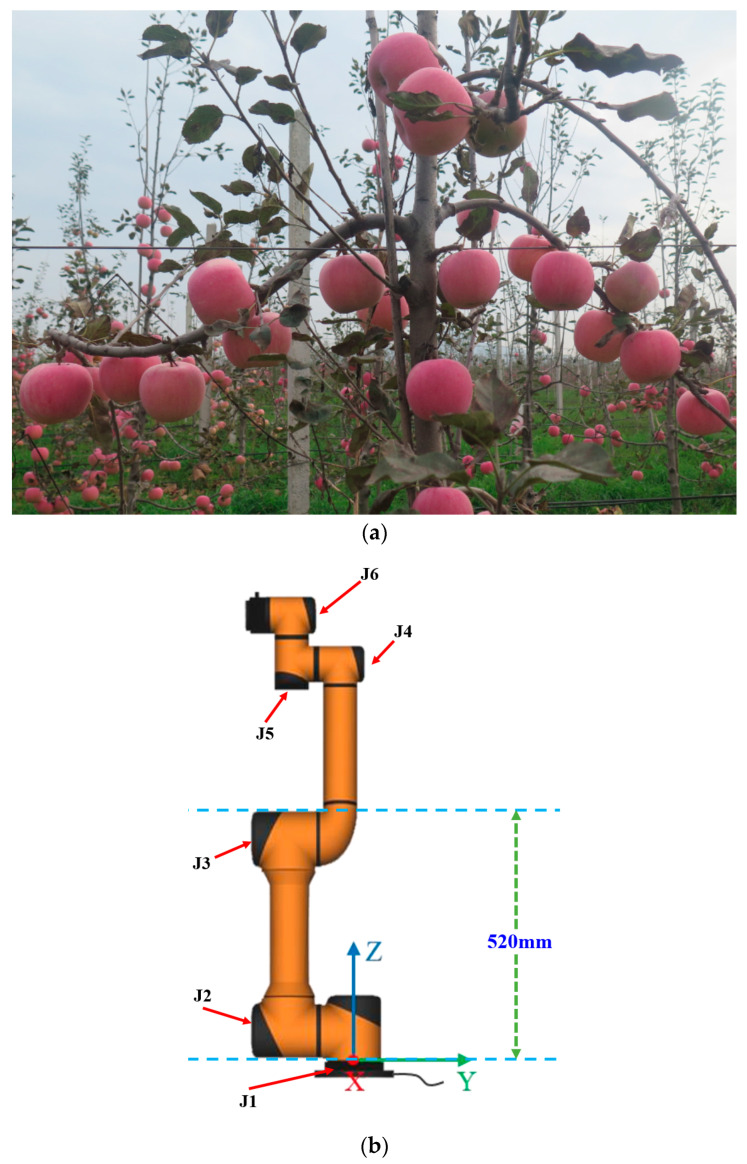
Spindle shaped apple tree (**a**) and core dimensions and joint diagrams of the AUBO-i5 manipulator (J_1_–J_6_ respectively represent the six joints of the robotic arm) (**b**). The study adopted an ‘overlapping workspace’ dual-arm layout. A front view schematic diagram of this layout is shown in Figure 2.

The AUBO-i5 manipulator (AUBO, Beijing, China) robotics technology Co., Ltd., Beijing, China, repetitive positioning accuracy: ±0.02 mm) [25,26,27,28,29] exhibits superior positioning accuracy and manipulation capabilities. Its exceptional motion control fidelity and adaptable six-degrees-of-freedom joint architecture facilitate accommodation of harvesting tasks across diverse elevations and orientations. The workspace of a dual-arm robot is formed by overlapping and combining two hemispherical workspaces with a radius of 886.5 mm. Payloads of 5 kg can be stably supported by this manipulator, sufficiently accommodating grasping requirements for various apple cultivars. Integrated safety systems—including impact sensing, joint torque surveillance, and instant halt functionality—effectively mitigate risks of damage to personnel or orchard assets, thereby satisfying collaborative operation standards. Employing modular architecture, the platform enables rapid component interchange and end-effector substitution, permitting adaptation to diverse harvesting implements (e.g., compliant grippers, vacuum-based adhesion units, and specialized harvesting end-effectors). Consequently, this research designated the AUBO-i5 as the foundational platform for constructing the dual-arm harvesting system. Figure 1 depicts spindle-structured apple trees alongside critical AUBO-i5 dimensional parameters.

The base origins for left and right manipulators are designated as O_1_ and O_2_, respectively, with O representing the central reference point. Segments OO_1_ and OO_2_ measure 550 mm, providing sufficient clearance for terminal tooling integration. Power interfaces for both manipulators are oriented externally relative to the diagrammatic plane.

**Figure 2:**
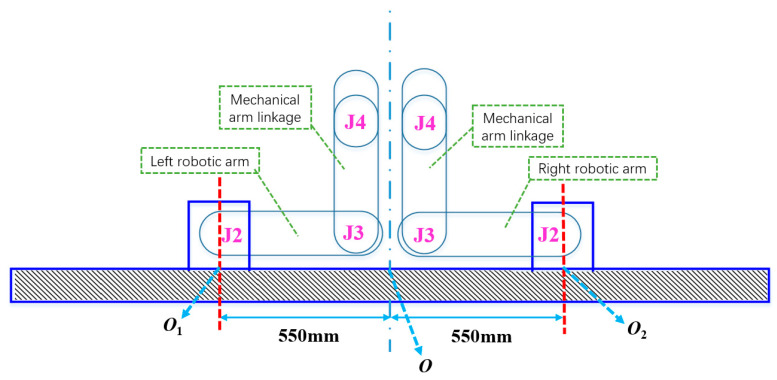
Schematic diagram of double-arm layout with overlapping working space (distance between left- and right-arm base centers = 1100 mm).

In the illustrated configuration, the left manipulator joints 2 and 3 were positioned at 90° rotational displacement, while the corresponding right manipulator joints were set to −90° orientation. Remaining joints maintained neutral (0°) alignment. Mechanical components J_2_–J_4_ with associated linkages are depicted in Figure 2.

Operational responsibilities were allocated such that left-sector harvesting targets were assigned to the left manipulator, while right-sector targets were managed by its counterpart. Visual-servo-constrained harvesting ensured collision-free terminal effector operation. Per AUBO-i5 specifications, the 1210 mm separation between base peripheries (2 × 605 mm) exceeds the cumulative linkage dimension for J_2_–J_3_ segments (1040 mm, Figure 2), thereby preventing mechanical interference between adjacent J_3_ assemblies.

Consequently, despite workspace overlap, visually constrained zone harvesting guarantees collision-free coordination.

**Figure 3:**
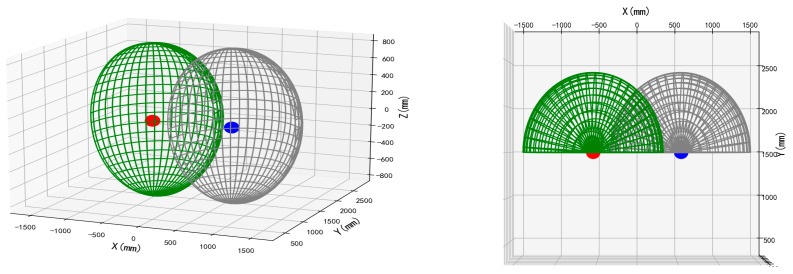
Schematic diagram of double-arm layout with overlapping working space.

For enhanced spatial analysis of the dual-manipulator arrangement, computational modeling was performed using Python (Version 3.7.10) to generate the overlapping workspace schematic (Figure 3). This representation comprises two partially superimposed hemispherical operational envelopes (radius: 886.5 mm), with O_1_ and O_2_ indicating base centroids.

**Figure 4:**
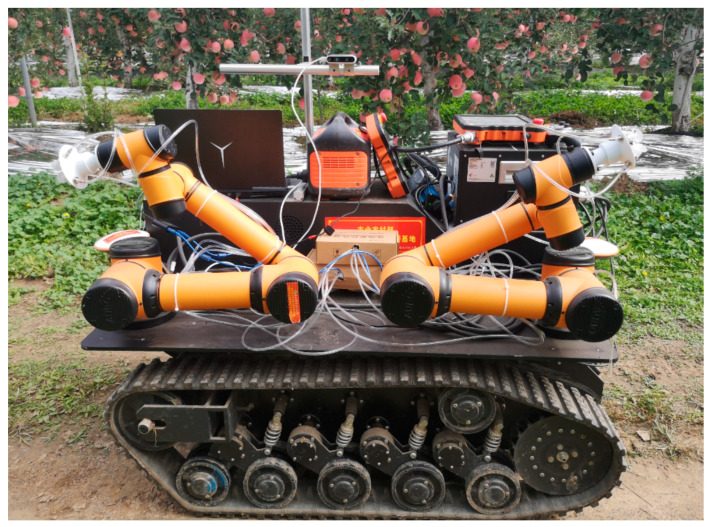
The prototype of intelligent fruit picking robot with double mechanical arms.

The physical drawing of the double manipulator built on the robot chassis based on this layout is shown in Figure 4. Dual robotic manipulators are incorporated: specifically, two AUBO-i5 lightweight collaborative arms possessing six degrees of freedom. A modular joint architecture is employed by the AUBO robotic arm, featuring a developer-centric operating system. Tailored robotic control systems designed for particular operational needs can be created by users, utilizing the application programming interfaces supplied by the AUBO framework. Furthermore, a dedicated programmable operator interface is integrated with the AUBO manipulator. Real-time operational data concerning the robot can be monitored through this interface, and diverse control configurations may be executed upon it.

The manipulator end-effectors are suction-based. Inspiration for this pneumatic biomimetic, non-destructive harvesting tool is derived from the mechanism employed by octopuses to capture prey. A symmetrical central arrangement characterizes the design, featuring four suction orifices uniformly positioned on the palmar section. Concave deformation conforming to the apple’s surface can be generated by the suction orifices under vacuum-induced negative pressure. Detachment of the apple from its branch is accomplished by the pulling force resulting from the negative pressure within the four orifices, thereby completing the harvesting task.

The vision subsystem integrates three primary components: a configurable imaging mount attached to the robotic control unit, an Intel Realsense D435 three-dimensional perception device, and its corresponding USB 3.2 data transfer conduit. Global shutter implementation and wide-angle coverage are featured in the D435 unit. Operational parameters include a near-field detection threshold of approximately 0.11 m and a far-field detection limit extending to 10 m, establishing suitability for modern standardized orchard configurations. Support for 1280 × 720 depth resolution enables effective deployment on automation platforms for acquiring spatial information regarding environmental targets.

Computational operations are hosted on a Lenovo Y7000P workstation serving as the primary hardware platform. Windows 10 ×64 functions as the operating environment. Processing tasks are managed by an Intel Core i7-10875H central processor. System memory employs DDR4 technology with 32 GB maximum capacity, while graphical computations are processed through an RTX 2060 GPU. Operational programs include visual recognition and localization algorithms; kinematic coordination protocols for dual AUBO-i5 manipulators; serial communication controllers (Zilog, Milpitas, CA, USA) for both compliant and vacuum-based end-effectors; and parallelized control software for simultaneous manipulator operation.

Regarding locomotion, wheeled propulsion systems were evaluated and discarded for orchard applications due to inadequate performance on compliant, irregular terrain. Tracked mobility platforms were subsequently selected, offering advantages including diminished turning circumference, reduced traction loss during movement, improved barrier traversal capability, and heightened environmental adaptability. Given the topographic complexity characterizing regional apple cultivation systems, tracked drivetrains were consequently designated as the propulsive foundation for harvesting automation.

### 2.2. Dynamic Zone Planning Method for Double-Arm Picking Operation Based on Pose Constraints

To enhance harvesting throughput in dual-manipulator systems, a zone partitioning methodology must be established to maximize synchronized parallelization, thereby reducing total operational duration. Computational assessment of displacement metrics is required for optimizing parallel task execution efficacy: (1) displacements between all target fruits and both terminal effectors should be quantified, and (2) cumulative trajectory lengths assigned to each manipulator must be aggregated. The allocation strategy necessitates minimal variance between aggregated displacements for left-zone apples relative to the left effector and corresponding measurements for right-zone apples relative to the right effector. This spatial equilibrium principle governs optimal zone distribution.

Because the design principle of the dynamic zoning method is different from the equal zoning method, the left and right boundaries of the dynamic zoning method are determined based on the spatial distribution of apples in the current robot field of vision, and the boundaries are not necessarily the opposite boundaries of the picking area. Therefore, it is very likely that the picking boundary will extend to the left or right across the equidistant boundary.

**Figure 5:**
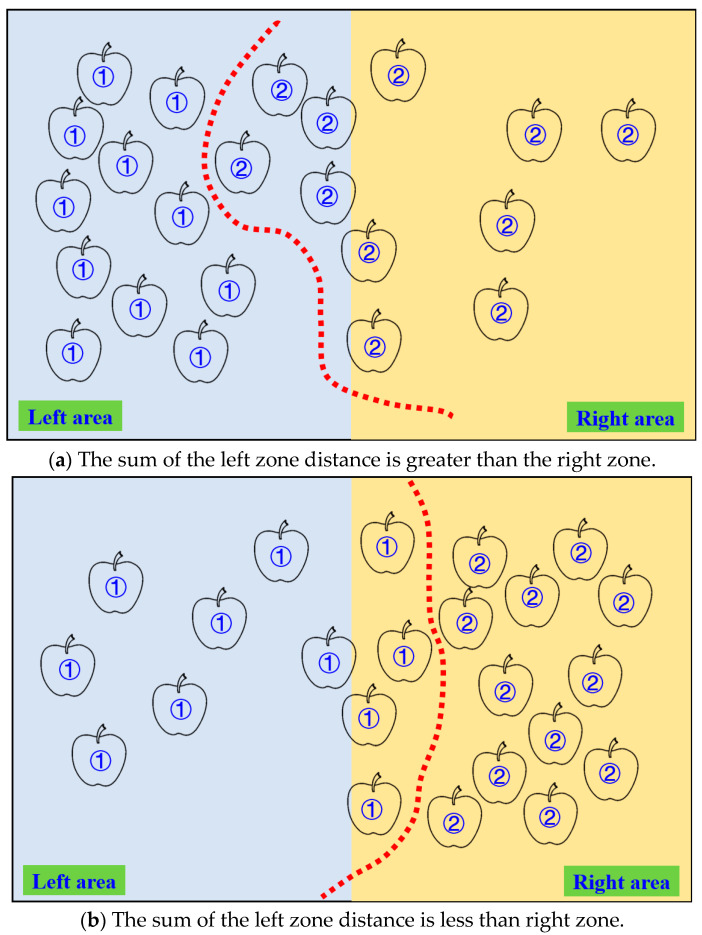
Schematic diagram of the dynamic zone planning method for double-arm picking.

The schematic diagram of the dual-arm dynamic picking-zone method is shown in Figure 5, including two cases: the left zone distance and greater than the right zone, the left zone distance and less than the right zone. The red dotted line is the dividing line of the dynamic partition. The apple numbered 1 is classified into the left zone after the dynamic partition algorithm, and the apple numbered 2 is classified into the right zone.

V-REP (Virtual Robot Experimentation Platform, Version 3.6.2) represents specialized software for simulating robotic systems, demonstrating particular strength in the kinematic analysis of robotic manipulators. Distributed control architecture can be implemented within this environment using embedded scripts, remote API interfaces, and similar mechanisms, rendering it an excellent resource for robotic simulation. Control algorithms enabling manipulator motion within the simulator can be authored in programming languages such as C/C++, Python, Java, Lua, MATLAB, Octave, or Urbi. Consequently, model visualization is facilitated, and the extraction of relevant simulation data parameters is enabled. The simulation phase is carried out to determine if the two arms can collide during operations.

**Figure 6:**
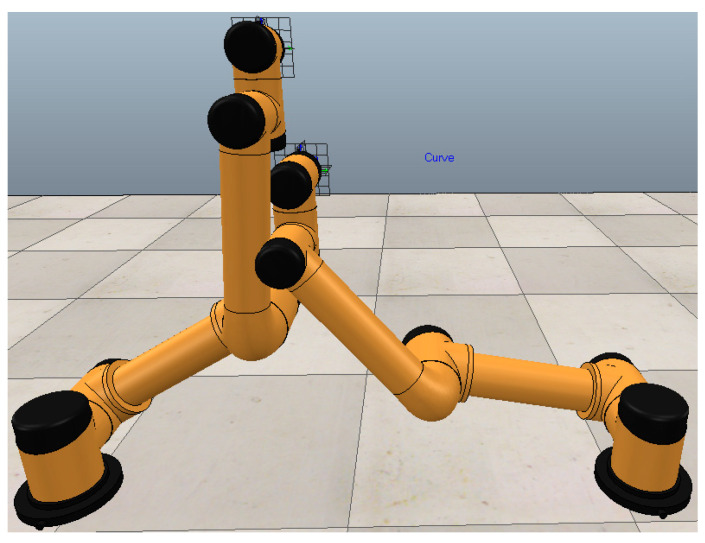
Schematic diagram of collision without posture constraint in dynamic zone planning for double-arm picking.

Because the left and right dividing line of the planned two-arm dynamic picking zones are likely to cross the equidistant line, the two arms may collide when the left and right mechanical arms cross the equidistant line to pick fruits. The simulation diagram of double-arm picking collision when the manipulator has no attitude constraint based on V-REP is shown in Figure 6. In the figure, the right manipulator crossed the dividing line and entered the left side for operation, and collided with the connecting rod between joints 3 and 4 of the left manipulator.

On the other hand, because the dynamic picking partition method considers the X coordinate size of the fruit centroid in the apple partition, based on the allocation of fruit partition by the robot vision system, there will be no collision between the ends of the left and right picking manipulator, and the collision phenomenon occurs between the end of one manipulator and the link or joint of the other manipulator (as shown in Figure 6 above), or between the link/joint of the left and right manipulator.

**Figure 7:**
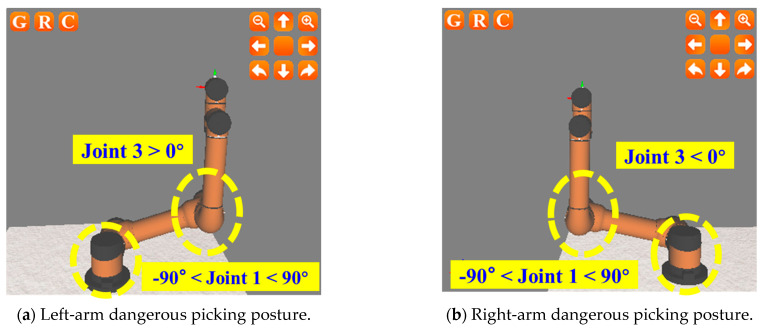

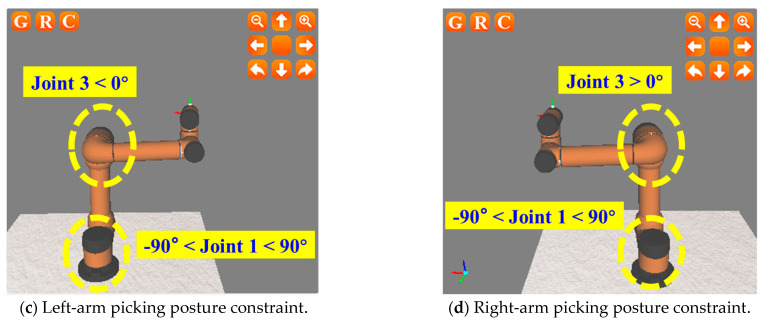
Dangerous picking posture and picking posture constraints of left and right arms.

Based on the analysis of the AUBO-i5 six-axis manipulator configuration, if the position and posture of the left and right manipulator in the picking process are constrained, the collision avoidance of both arms in the dynamic picking partition method can be guaranteed. Among them, the picking pose constraints of the manipulator for double-arm collision avoidance mainly involve joint 1 and joint 3 of the manipulator. The specific picking constraint posture of the left and right manipulator is analyzed as follows:

For the left manipulator: when joint 1 is in the range of (−90°, 90°), the picking posture of joint 3 > 0° is a dangerous posture, which easily leads to the collision of both arms (as shown in Figure 7a); when joint 1 is in the range of (−90°, 90°), the picking posture of joint 3 < 0° is a safe posture (as shown in Figure 7c).

For the right manipulator: when joint 1 is in the range of (−90°, 90°), the picking posture of joint 3 < 0° is a dangerous posture, which easily leads to the collision of both arms (as shown in Figure 7b); when joint 1 is in the range of (−90°, 90°), the picking posture of joint 3 > 0° is a safe posture (as shown in Figure 7d). The analysis of the dangerous/safe picking constraint posture for the left and right manipulators is shown in Table 1.

Based on the examination of safety constraints for harvesting postures in both manipulators, the preparatory stance for dual-arm fruit collection is defined. Direct movement from the fruit deposition orientation to the apple’s location would cause the robotic arm to encounter interference from arboreal structures; consequently, essential movement constraints must be imposed during the approach and harvesting phases. A predefined preparatory configuration is therefore established—specifically, an intermediary configuration preceding fruit acquisition that must be attained following fruit placement. This ensures harvesting success rates.

**Figure 8:**
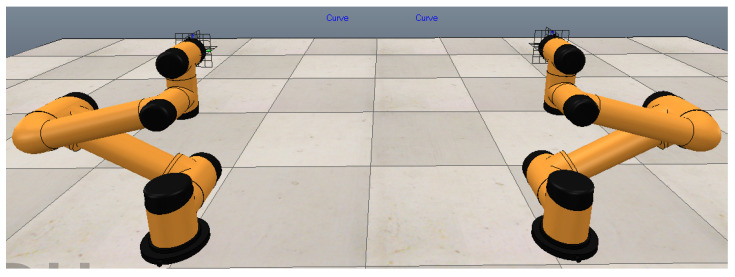
Simulation diagram of the double-arm picking-preparation posture constraint based on the dynamic zoning method.

Within the dynamic zoning methodology, the end-effector position coordinates (X, Y) in this preparatory stance are configured to match the center coordinates of the manipulator base. The Z-coordinate is set at 50% of the end-effector’s height in the initial vertical orientation (492.5 mm). Crucially, angular limitations for joint 3 differ between manipulators in this preparatory state: the left arm maintains joint 3 below 0°, while the right arm requires joint 3 above 0°. The simulated preparatory configuration for bimanual apple harvesting is illustrated in Figure 8.

**Figure 9:**
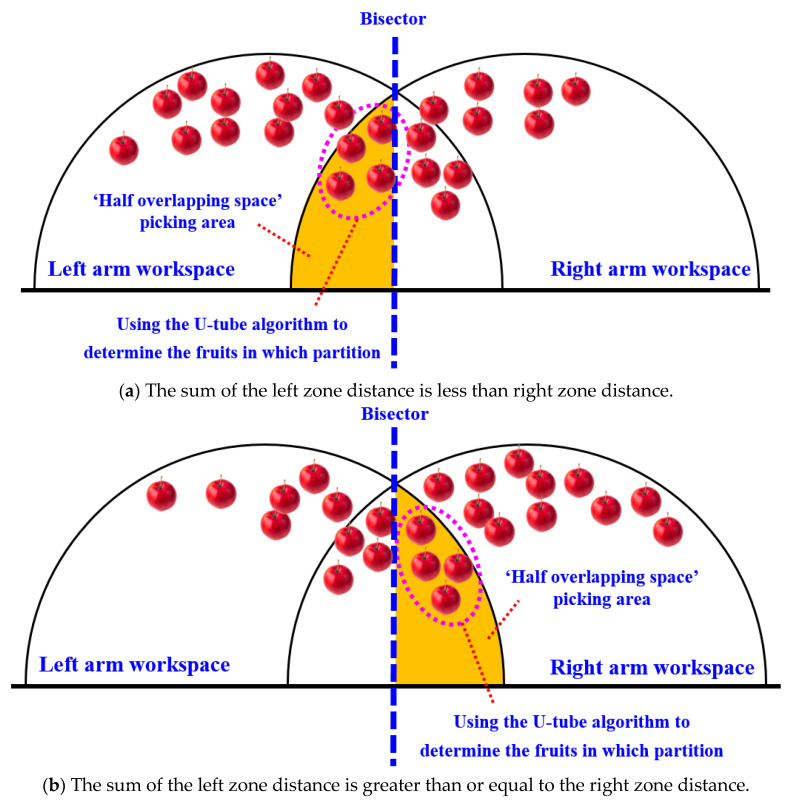
Top view of the dynamic zoning situation for double-arm picking.

Based on the size parameters of the AUBO-i5 manipulator, the dynamic zoning method of double-arm picking was further analyzed. The top view of dynamic zoning is shown in Figure 9.

When the sum distance of the left zone is less than the right zone, in order to make the double manipulator work in parallel and at the same time as much as possible, the right manipulator needs to cross the dividing line and enter the left side for picking.

On the other hand, restricted by the arm length of AUBO-i5 manipulator, the right manipulator can only pick fruits within the hemispherical picking range of the right arm on the right side of the dividing line and the hemispherical picking range of the right arm on the left side of the dividing line (as shown in the orange area in Figure 9a). Therefore, when the distance between the left zone and the right zone is less than the distance between the left zone and the right zone, for all apples within the picking range of the dual-arm robot, the fruits on the right side of the peer line can be directly allocated to the right arm for picking, and the fruits on the left side of the peer line and outside the hemispherical working space of the right arm can be directly allocated to the left arm for picking.

In addition, it is also necessary to plan for picking the fruits in the orange zone (the ‘semi-overlapping space’ picking area marked in orange in Figure 9a). The planning follows the principle of ‘making the distance and proximity between the left and right zones as far as possible’.

When the sum distance of the left zone is greater than or equal to the right zone, for all apples within the picking range of the dual-arm robot, the fruits on the left side of the peer line can be directly allocated to the left arm for picking, and the fruits on the right side of the peer line and outside the left-arm hemispherical working space can be directly allocated to the right arm for picking.

In addition, it is also necessary to plan for the fruits in the orange zone (the ‘semi-overlapping space’ picking area marked in orange in Figure 9b). The planning also follows the principle of ‘making the distance and proximity between the left and right zones as close as possible’.

In order to optimize the partition allocation of apples in the picking area of the ‘semi-overlapping space’ in Figure 9, so as to achieve ‘the distance and proximity of the left and right areas as far as possible’, a ‘U-tube optimal allocation algorithm’ was proposed. The mathematical expression of the objective function of the algorithm is shown in Equations (1) and (2). The goal of the optimal allocation algorithm is to automatically calculate and obtain the zoning plan for apples within the dual-arm picking range that minimizes the value of the objective function.

(1) $$E_{min}=\left|\left(D_{l}+\sum_{1}^{i}L_{i}\right)-\left(D_{r}+\sum_{i+1}^{m}R_{n}\right)\right|\;\;\;\;\;\;\;\;\;(DL_{all}\geq DR_{all})$$

(2) $$E_{min}=\left|\left(D_{l}+\sum_{i+1}^{m}L_{n}\right)-\left(D_{r}+\sum_{1}^{i}R_{i}\right)\right|\;\;\;\;\;\;\;\;\;(DL_{all}<DR_{all})$$

In the formula, when the distance between the apples to be picked within the picking range of the dual-arm robot and the end of the left arm and *DL_all_* is greater than or equal to the distance between the apples to be picked and the end of the right arm and *DR_all_*, then the variables are defined as follows:

*D_l_* represents the distance between all fruits located within the picking range of the left arm on the left side of the equivalence line and the end of the left arm.

*D_r_* represents the distance between all fruits located on the right side of the equivalence line and outside the working space of the left arm and the end of the right arm.

*L*_1_, *L*_2_…*L_i_* represent the distances between the apples picked by the left arm from the ‘semi-overlapping space’ picking area on the right side of the equivalence line and the end of the left arm.

*R_i+_*_1_…*R_m_* represent the distances between the apples picked by the right arm from the ’semi-overlapping space‘ picking area on the right side of the equivalence line and the end of the right arm.

**Figure 10:**
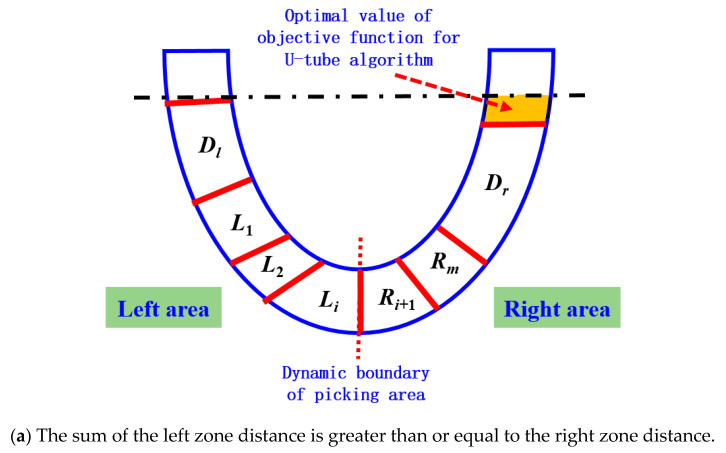

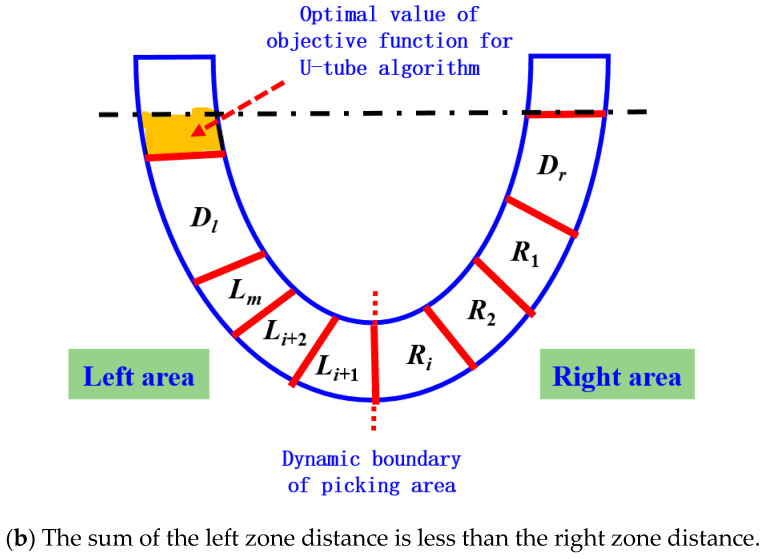
Schematic diagram showing the principle of the U-shaped tube optimal allocation algorithm.

*E_min_* represents the optimal value of the objective function of the ‘U-tube optimal allocation algorithm’ (as shown in the orange area in Figure 10), and the smaller the value of the objective function, the better the performance of dynamic partition programming.

When the distance between the apples to be picked within the picking range of the dual-arm robot and the end of the left arm, *DL_all_*, is less than the distance between the apples to be picked and the end of the right arm, *DR_all_*, then the variables are defined as follows:

*D_r_* represents the distance between all fruits within the picking range of the right arm on the equal line and the end of the right arm.

*D_l_* represents the distance between all fruits located on the left side of the equal line and outside the working space of the right arm and the end of the left arm.

*R*_1_, *R*_2_…*R_i_*, respectively, represent the distance between the apples to be picked by the right arm from the ’semi-overlapping space‘ picking area on the left side of the equal line and the end of the right arm.

*L_i+_*_1_, *L_i+_*_2_…*L_m_*, respectively, represent the distance between the apples to be picked by the left arm from the ‘semi-overlapping space’ picking area on the left side of the equal line and the end of the left arm. The orange area in Figure 10 represents the optimal value (*E_min_*) of the objective function of the ‘U-tube optimal allocation algorithm’.

The schematic diagram showing the principle of the ‘U-shaped tube optimal allocation algorithm’ is shown in Figure 10.

Taking into account factors such as the overlapping layout of the dual-arm workspace, constraints on the picking range of the left and right arms, and constraints on the picking pose, a dynamic partition planning method for dual-arm picking is proposed. The main steps are as follows:

(1) Activate the vision system of the dual-arm picking robot: Start the apple target recognition and positioning program (YOLO-based artificial intelligent detection network for apple targets [7]) on the robot control terminal, enable real-time fruit target detection, and determine whether there are apple targets to be picked in the space that can be picked by both arms. If there are any, proceed to the next step. Otherwise, keep both arms in a standby state and wait for a target fruit that can be picked to appear in the field of view.

(2) Obtain the x-coordinate value of the center of mass of the fruit: Based on the visual system, obtain the x-coordinate value in the camera coordinate system for the apple target within the range that can be picked by both arms.

(3) Calculate d-left arm and d-right arm separately: Based on the positioning results of the visual system, calculate the distance d between each apple within the picking range and the left-arm end-effector, and the distance d between each apple and the right-arm end-effector.

(4) Calculate *DL_all_* and *DR_all_* separately: Based on the d-left and d-right arms of each apple obtained in (3), calculate the sum of all d-left arms as *DL_all_* and all d-right arms as *DR_all_*.

(5) According to the size of *DL_all_* and *DR_all_*, different partitioning methods are adopted for the fruits:

If *DL_all_* < *DR_all_*: ① All fruits on the right side of the equivalence line will be assigned to the right area, and the right robotic arm will be responsible for picking them. ② Assign fruits within the left-arm picking range and outside the right-arm picking range to the left area, and have the left robotic arm responsible for picking them. ③ Calculate the d-right arm of the fruit within the picking range on the right arm of the equivalent line. ④ Calculate the d-left arm of the apple within the left-arm picking range and outside the right-arm picking range. ⑤ Sort the fruits within the picking range of the right arm on the left side of the contour line in order from right to left based on the x-coordinate value of the fruit parenchyma center (in ascending order of d-right arm for the same x-value). ⑥ Based on the ‘U-shaped tube optimal average allocation algorithm’, calculate separately the optimal attribution partition for the fruits involved in step ⑤. ⑦ When the objective function value of the U-tube algorithm is minimized, it is the optimal partition, and the dynamic partition result of the current apple to be harvested is obtained.

If *DL_all_* ≥ *DR_all_*: ① All fruits on the left side of the equivalence line will be assigned to the left area, and the left robotic arm will be responsible for picking them. ② Allocate fruits within the right-arm picking range and outside the left-arm picking range to the right area, which is responsible for picking by the right robotic arm. ③ Calculate the d-left arm of the fruit within the picking range on the left arm of the equivalent line. ④ Calculate the d-right arm of apples within the right-arm picking range and outside the left-arm picking range. ⑤ Sort the fruits within the picking range of the left arm on the right side of the contour line in order from left to right based on the x-coordinate value of the fruit parenchyma center (in ascending order of the d-left arm for the same x-value). ⑥ Based on the ‘U-shaped tube optimal average allocation algorithm’, calculate separately the optimal attribution partition for the fruits involved in step ⑤. ⑦ When the objective function value of the U-tube algorithm is minimized, it is the optimal partition, and the dynamic partition result of the current apple to be harvested is obtained.

(6) Drive the left and right robotic arms to their respective picking constraint positions.

(7) According to the dynamic partition planning method for dual-arm picking, the apple partitioning results are obtained, and the left and right arms, respectively, perform the picking operation.

(8) If all planned fruits have been harvested, return to step (1), otherwise continue with the harvesting operation.

**Figure 11:**
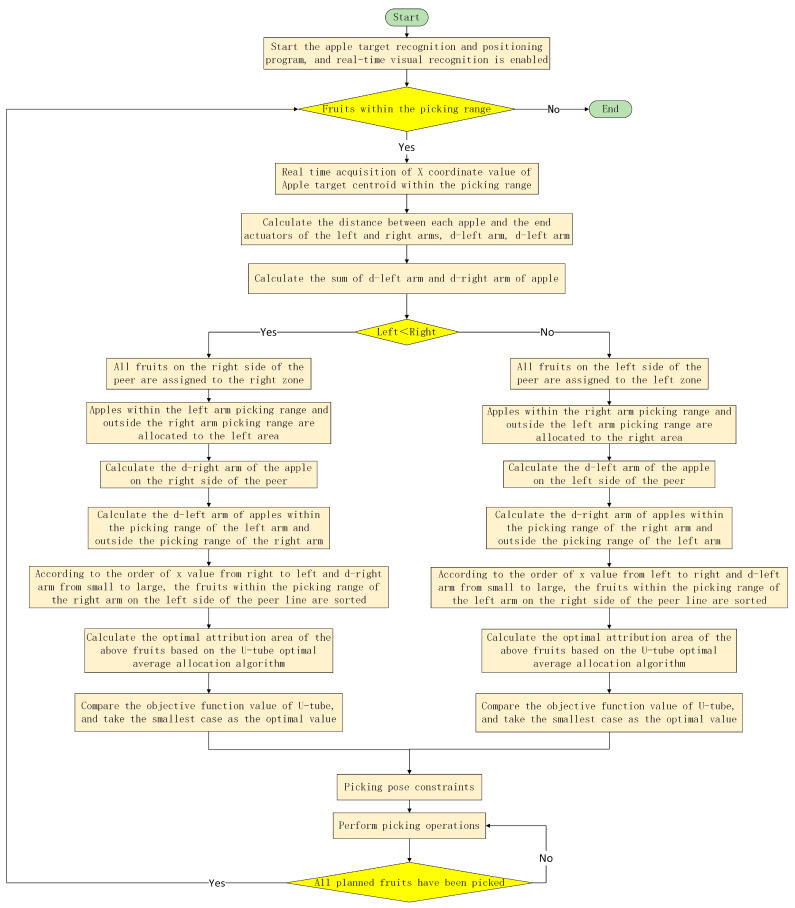
Picking process of dynamic zone picking method.

The picking process based on the dynamic partitioning method of the dual-arm picking operation is shown in Figure 11.

**Figure 12:**
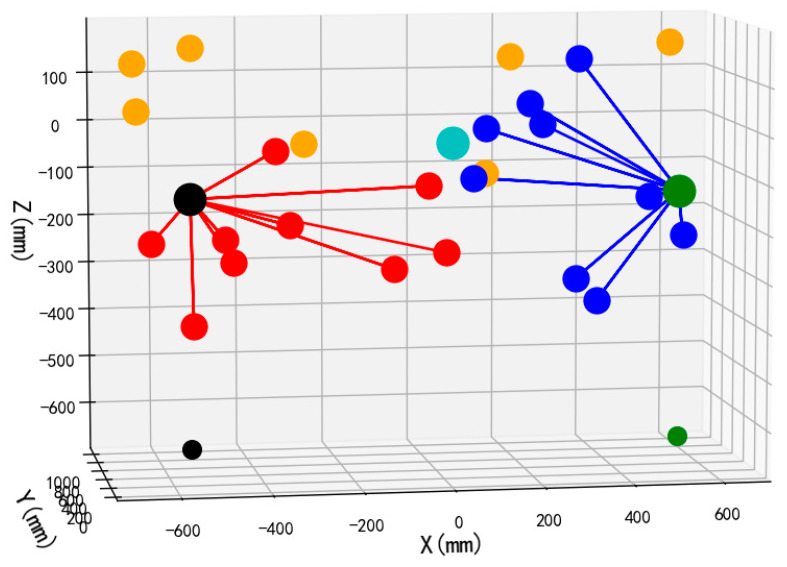
Schematic diagram of the double-arm fruit picking task distribution based on dynamic zoning.

To validate the performance and operational efficacy of the suggested bimanual harvesting dynamic partitioning algorithm within robotic applications, the approach was programmed in Python and fruit traversal simulations were performed. Prior to configuring the simulation trials, spatial coordinates for critical robotic hardware components were established. These included the base centers of both harvesting manipulators, the mounting location of the D435 depth camera, and the end-effector positions for the left and right arms. Distinctive colored markers were applied to essential robotic elements, with the simulation layout depicted in Figure 12. Crimson markers indicate apples positioned within the left operational zone, azure markers signify fruit located in the right sector, and amber markers represent targets beyond the bimanual harvesting range.

Commencing each harvesting operation from the preparatory stance, the robotic arm initiates apple collection and must revert to this configuration following fruit release before subsequent harvesting. Consequently, the manipulator’s harvesting trajectory can be approximated by the Euclidean distance between targeted fruit and the end-effector in the preparatory configuration. For both manipulators, cumulative linear distances between partitioned apples and their respective end-effectors in preparatory configurations were computed following dynamic partitioning, yielding total operational path lengths for each arm.

Validation of the proposed bimanual harvesting dynamic partitioning method—incorporating pose constraints for collision avoidance—was performed through partitioning simulations utilizing stochastically distributed target coordinates. These simulations were constructed within a MATLAB environment (Figure 12), with three-dimensional positional data for harvested apples recorded separately for each manipulator. Vermilion markers denote left-arm harvested apples, cerulean markers indicate right-arm harvested targets, and amber markers represent unharvested fruit beyond the bimanual operational range.

**Figure 13:**
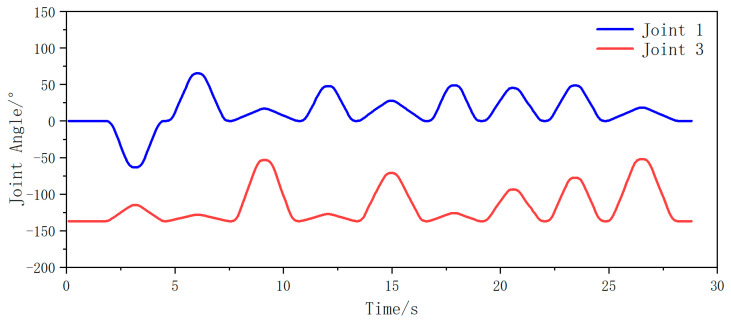
Changes in angle values of joints 1 and 3 during iteration of the left arm through the fruits in left area.

**Figure 14:**
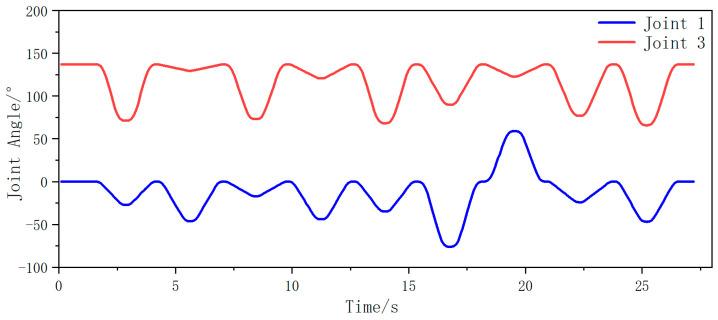
Changes in angle values of joints 1 and 3 during iteration of the right arm through the fruits in right area.

Subsequently, joint angle variations between joint 1 and joint 3 were captured during biaxial motion as both AUBO-i5 manipulators traversed their coordinate sequences. Temporal changes in these joint angles are illustrated in Figure 13 (left manipulator) and Figure 14 (right manipulator).

From the analysis in the figure above, it can be seen that during the entire fruit traversal motion of both arms, the angle of joint 1 is always within the range of (−90°, 90°) for the left robotic arm, and the angle of joint 3 is always within the range of less than 0°. Therefore, the motion posture of the left arm is within the angle constraint range of the safe posture. For the right robotic arm, the angle of joint 1 is always within the range of (−90°, 90°), and the angle of joint 3 is always within the range greater than 0°. Therefore, the motion posture of the right arm is also within the angle constraint range of the safe posture. In summary, in the picking traversal workflow based on dynamic partitioning, the motion posture of both arms is within the safe range of pose constraints, and there will be no collision phenomenon.

To enable deeper assessment of the bimanual dynamic zoning algorithm’s efficacy, four experimental series evaluating the dynamic zoning methodology were executed. Each series incorporated two distinct configurations: one where cumulative proximity values for the left manipulator relative to targets in its zone exceeded or matched the corresponding values for the right manipulator (i.e., collective distances from harvestable apples to the left end-effector were greater than or equal to those to the right end-effector), and another configuration where the left manipulator’s cumulative proximity was less than or equal to the right manipulator’s equivalent measurements.

**Figure 15:**
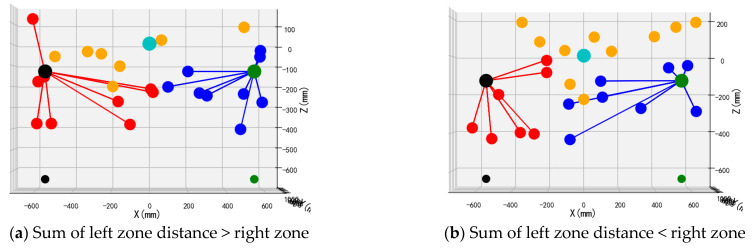
Simulation test 1.

**Figure 16:**
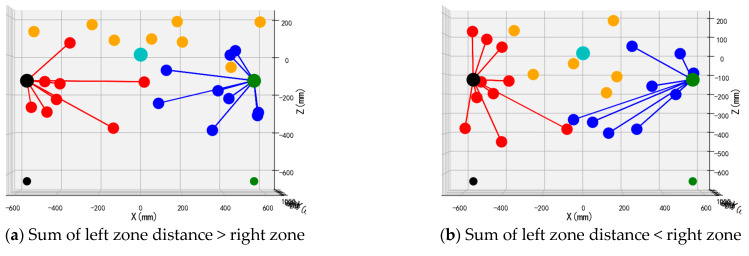
Simulation test 2.

**Figure 17:**
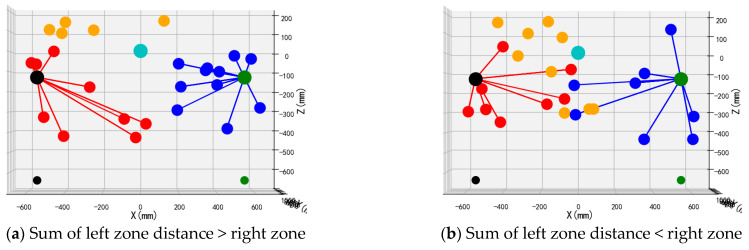
Simulation test 3.

**Figure 18:**
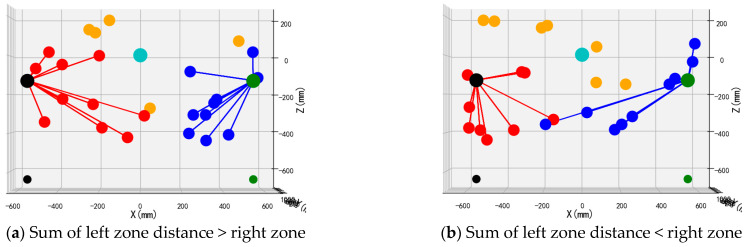
Simulation test 4.

The front views of the 3D schematic diagrams of four sets of simulation experiments for the dynamic partition planning method of dual-arm picking based on pose constraints are shown in Figure 15, Figure 16, Figure 17 and Figure 18. Vermilion spheres denote targets allocated to the left operational zone, cerulean markers identify fruit positions assigned to the right sector, and amber markers designate unreachable targets excluded from bimanual harvesting.

Simulation outcomes corresponding to dynamic zoning for bimanual harvesting are presented in Table 2. For every experimental configuration under distinct fruit distributions, multiple parameters were documented: the quantity distribution between left and right operational zones; targets situated within partially overlapping regions; traversal path lengths for both manipulators; aggregate bimanual traversal distance; peak single-manipulator traversal length; and maximum concurrency ratio during coordinated operations.

The tabulated data reveal that across four simulation trials, peak concurrency proportions for coordinated manipulator operations ranged from 82.1% to 99%. Extreme traversal distances between 4404 mm and 6537 mm for individual manipulators were documented, while aggregate bimanual path lengths spanned from 9237 mm to 11,904 mm.

The performance variations observed across the four test groups primarily stem from differences in spatial fruit distribution and the adaptive response of the U-tube optimization-based zoning and sequencing strategy. In groups where fruit distribution was more balanced between left and right zones—particularly when the semi-overlapping space contained an adequate number of fruits—the system achieved higher parallel operation ratios (e.g., 99% in groups 2 and 4) and more symmetric arm travel distances, indicating efficient dynamic task allocation and reduced idle time. Conversely, in less balanced scenarios, such as group 3 with left zone ≥ right zone, where one zone contained significantly more fruits and the overlapping region was limited, the parallel ratio decreased to 82.1% and path lengths became uneven, reflecting a natural adjustment of the algorithm to prioritize collision-free operation over ideal parallelism. Despite these variations, the algorithm consistently minimized total travel distance (e.g., 9237 mm in group 1 under left zone < right zone) and maintained collision-free operation across all cases, demonstrating its robustness in adapting to varying orchard conditions while ensuring operational efficiency and safety.

Applying the principle of ‘maximizing the distance and proximity between the left and right zones’ in the picking dynamic partition planning method, the task allocation for fruits within the dual-arm picking range is carried out. Consequently, regarding harvesting efficiency, maximization of concurrent manipulator utilization is achieved, ensuring peak bimanual coordination ratios and enhancing overall equipment productivity. However, owing to demarcation boundaries being determined by fruit spatial distributions within the current observational scope, harvesting zones frequently extend asymmetrically beyond the central division. This may allocate targets within partially overlapping harvest regions to more distant manipulators, indicating individual apples are not invariably assigned to the nearest available arm. Thus, the aggregate traversal distance for completing harvesting tasks becomes suboptimal—specifically, minimal trajectory requirements for bimanual apple retrieval are not guaranteed—resulting in elevated kinetic expenditure during robotic harvesting operations.

### 2.3. Fruit Picking Sequence Planning Method for Dynamic Partitioning of Dual-Arm Picking

The sequencing methodology for dynamically zoned harvesting prioritizes proximal targets within assigned sectors, adhering to the principle of harvesting fruits nearest to the manipulator’s end-effector first. Prioritized sequences for left/right sectors are established by ordering targets according to ascending Euclidean distances from each apple to the corresponding manipulator’s end-effector. The computational formulation for linear distance *L_apple_* between fruit targets and end-effectors remains consistent with prior formulations.

**Figure 19:**
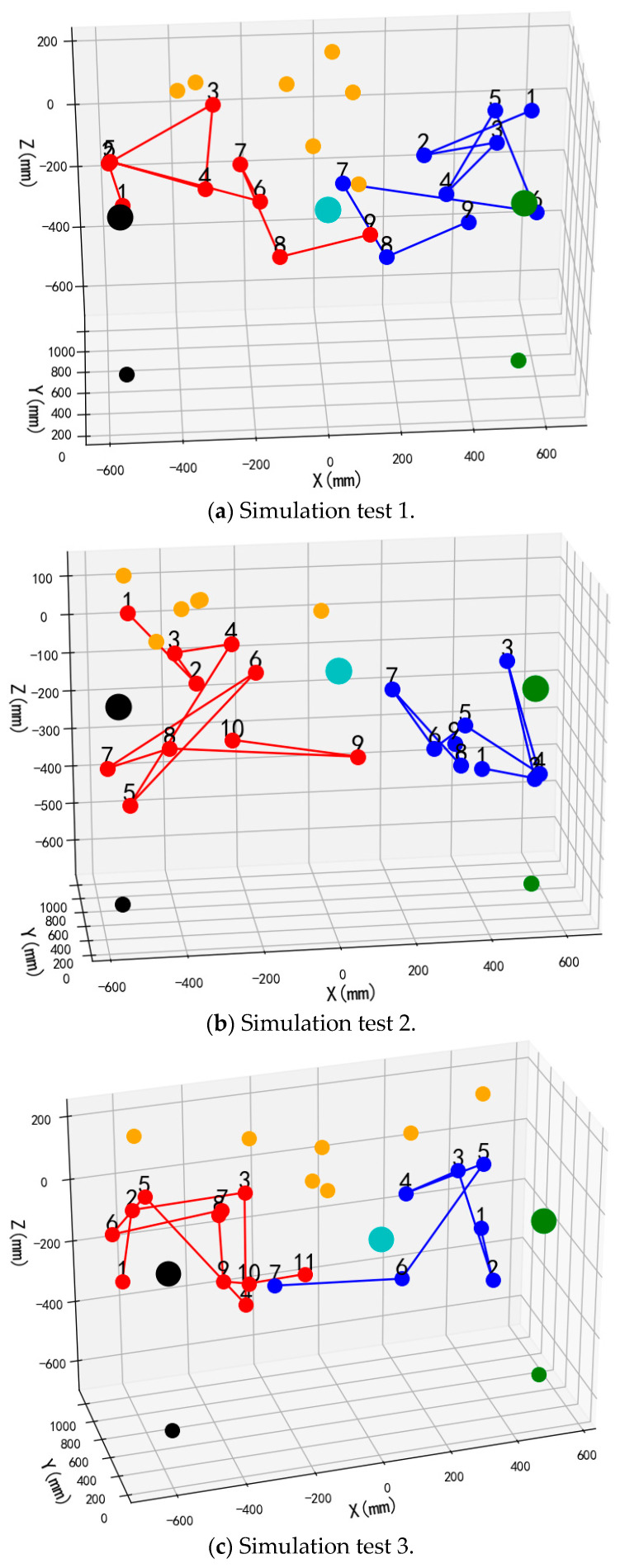
Simulation test of dynamic zone picking sequence planning.

Validation of the proposed dynamic zone sequencing approach for bimanual operations was executed through harvesting sequence simulations. Following Python-implemented dynamic zoning, traversal experiments were performed based on the proximal harvesting priority principle. Three experimental trials utilizing stochastically distributed spatial coordinates were conducted, with the simulation layout depicted in Figure 19. Sequentially numbered harvest indices are automatically annotated above targets, while manipulator trajectories following the harvesting sequence are visually indicated. Vermilion spheres designate left-arm harvesting locations and azure markers signify right-arm collection sites.

**Figure 20:**
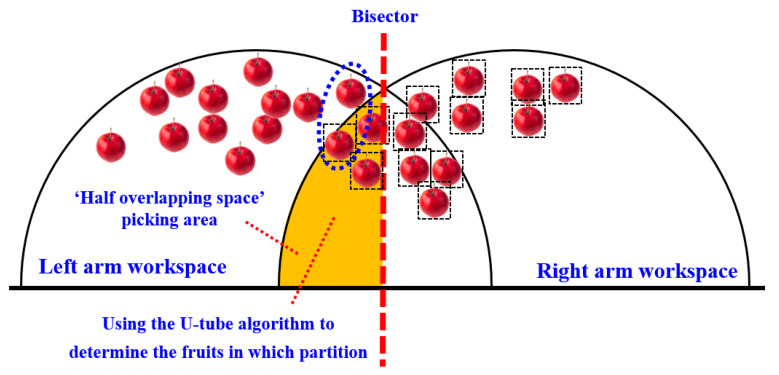
Top view of A type situation of dynamic picking zone.

Within the dynamic zone methodology, manipulators may traverse the central demarcation axis during harvesting activities. Considering the hemispherical configuration of the operational envelope, a particular operational scenario requires attention during bimanual harvesting applications: when the right manipulator crosses into left-partition territory, spatial coordinates of apples within the left operational zone but outside the right manipulator’s reach may exhibit x-values exceeding those assigned to right-manipulated targets in partially overlapping regions. This spatial anomaly is visually demonstrated in Figure 20, where two such targets are circumscribed by azure dashed contours (with right-manipulator harvested apples demarcated by obsidian dashed enclosures). Therefore, simultaneously picking of these two fruits with both arms may lead to collision and conflict between the robotic arms.

**Figure 21:**
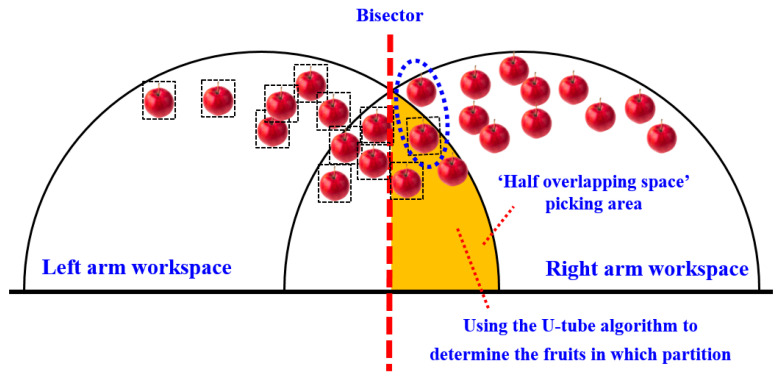
Top view of B type situation of dynamic picking zone.

Symmetrically, when the left manipulator enters the operational territory beyond the partition boundary, a comparable phenomenon may manifest: x-values of targets harvested by the right manipulator within its hemispherical harvesting volume—yet exterior to the left-manipulator’s operational range—might surpass the x-coordinates allocated to the left-manipulator-collected fruits in partially overlapping zones. This spatial configuration is illustrated in Figure 21, where two relevant targets are encircled with cyan dotted perimeters (left-manipulator-harvested fruits being demarcated using onyx dotted enclosures). Simultaneous picking of these two fruits by both arms may also cause collision and conflict between the robotic arms.

Consequently, a programmatic methodology was implemented within this investigation to mitigate potential interference hazards: during dynamic zone harvesting execution, x-coordinate values for targeted fruits are recorded as isolated variables prior to subsequent harvesting operations (with the coordinate system’s origin established at the midpoint between the manipulator bases). Following each harvesting action, these parameters are refreshed accordingly.

Before performing the picking operation on each fruit, the robotic arm needs to compare the x-coordinate values of the two fruits stored in the left and right arms. Owing to potential asynchronicity in harvesting cycles between the manipulators, execution may be temporarily suspended when positional conflicts are detected (specifically, if the x-coordinate of a left-allocated target is inferior to its right-allocated counterpart). The non-operational manipulator remains in standby until its counterpart completes the current harvesting sequence, updates stored target coordinates, and satisfies the conditional requirement where left-allocated targets exhibit superior x-values relative to right-allocated targets. Subsequently, harvesting operations are resumed by the previously idled manipulator.

**Figure 22:**
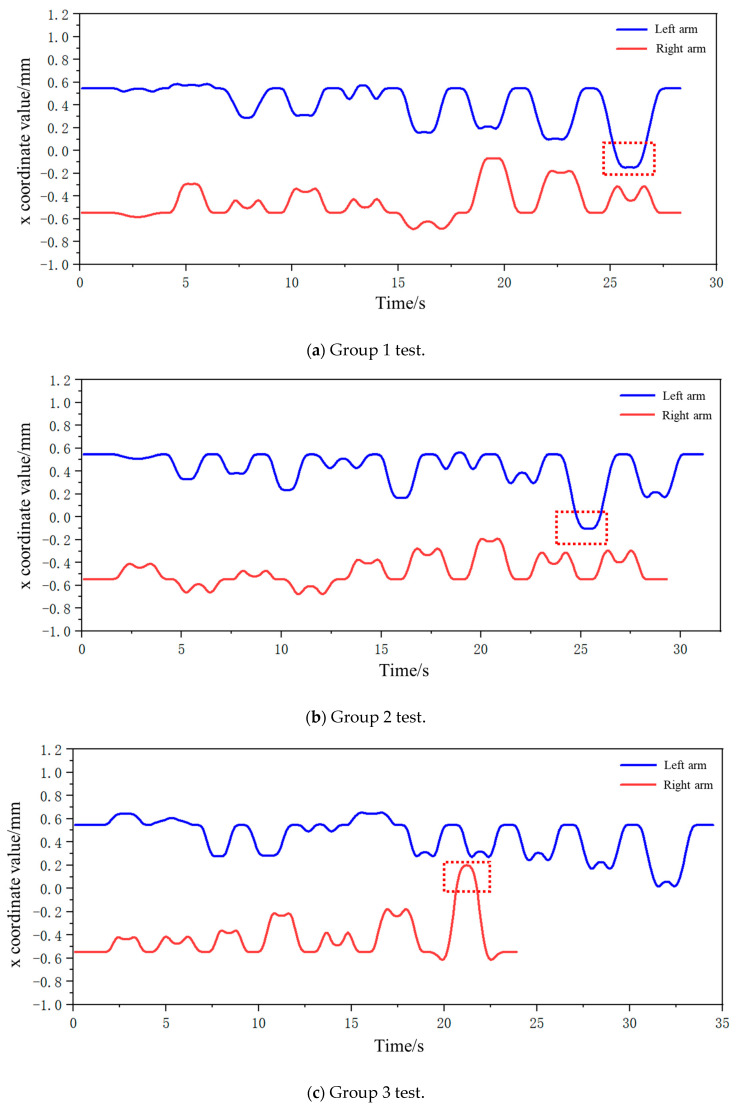
Changes in the x-coordinate values of the left and right mechanical-arm end-effectors in the simulation test.

Temporal variations in end-effector x-coordinates across three experimental trials are illustrated in Figure 22a–c. Commencing from dynamically zoned preparatory configurations, both manipulators sequentially attain target coordinates before reverting to preparatory stances prior to subsequent target acquisition. Critical observations reveal that although transgression beyond the x = 0 demarcation boundary occurs (denoted by vermilion dashed enclosures in Figure 22), trajectory profiles demonstrate non-intersecting characteristics. This absence of path intersection indicates collision-free bimanual operation throughout the harvesting sequence, thereby validating the safety and operational reliability of the proposed dynamic zone methodology.

## 3. Results and Discussion

**Figure 23:**
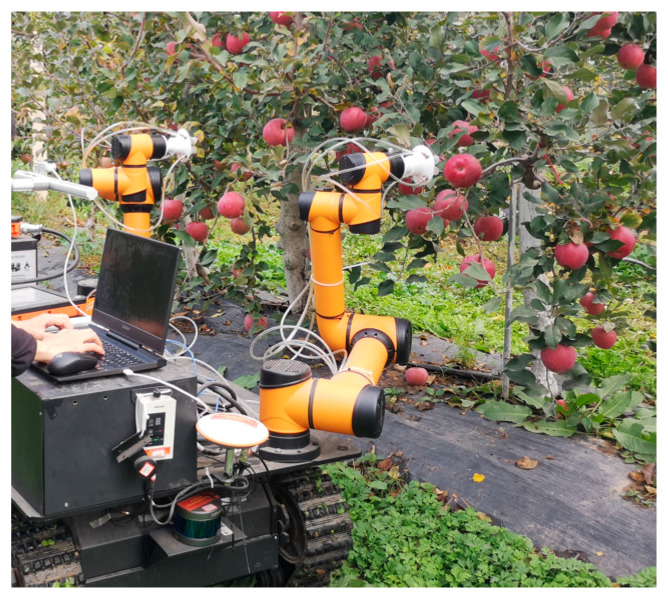
Scene of the dual-arm picking robot working in an orchard.

Validation trials employing bimanual manipulator systems for orchard applications were executed. Field testing of the robotic platform, implementing the proposed harvesting zone methodology, was conducted within a modern apple research station in Baishui County, Weinan City, Shaanxi Province (35° N, 109° E). Experiments were performed during phenological maturity of espalier-trained Red Fuji cultivars in high-density dwarf rootstock plantings. All evaluated fruits exhibited optimal growth parameters under natural conditions. To isolate experimental variables, pedicel-abscission interventions were omitted; exclusively spatial coordinates within predefined operational boundaries were traversed. This methodology facilitated verification and analysis of algorithmic performance for the zone planning approach integrated into the bimanual robotic system. The orchard validation environment is depicted in Figure 23.

A total of four sets of orchard validation tests for robotic operation efficiency were conducted in a standard orchard. In each test set, fruit traversal harvesting operations were performed using the ‘Pose-Constrained Dual-Arm Harvesting Dynamic Partition Planning Method’. Apple traversal sequencing adhered to the harvesting prioritization methodology introduced in this research. During experimental procedures, both manipulators initiated operations from dynamically assigned preparatory configurations. Following displacement to a target fruit’s coordinates, each manipulator reverted to its harvesting stance through articulated maneuvers prior to subsequent target acquisition. Outcomes from four orchard trials are compiled in Table 3.

Furthermore, the metrics denoted as ‘traversal length’, ‘operation path length’, ‘task length’, and ‘traversal path length’ uniformly represent trajectory distance (mm) traversed by AUBO-i5 end-effectors (designated as harvesting manipulators).

The concurrency ratio metric serves as an indicator of robotic operational efficiency, where elevated parallelization correlates with enhanced aggregate harvesting throughput and improved equipment utilization. For the adaptive zoning methodology, bimanual task concurrency ratios range from 85.7% (minimum) to 93.3% (maximum).

Total traversal duration reflects temporal requirements for completing all zoned harvesting assignments, providing supplementary efficiency assessment. From this perspective, the robotic system employing dynamic zoning exhibits traversal times spanning 17.8 s (shortest) to 22.3 s (longest).

Path differentials between manipulators under this methodology demonstrate variations from 267 mm (minimal) to 541 mm (maximal). Additionally, fruit allocation discrepancies between operational zones show a null differential at minimum and unity differential at maximum. Consequently, enhanced distributional equilibrium is achieved through implementation of the dynamic zone approach.

Compared with the equal partition planning method of dual robotic arms [30], in the context of identical apple harvesting scenarios, a greater rate of parallel operation for dual arms is observed when the dynamic partition planning approach is employed, as opposed to the equal partition method. This measurement serves as an indicator of robotic parallel task efficiency, where an increased proportion of concurrent operation correlates with enhanced overall harvesting performance, thereby implying improved utilization of the entire robotic system. More precisely, the parallel operation rate for dual arms utilizing dynamic partition planning is recorded to vary between 85.7% and 93.3%, whereas the range for the equal partition method falls from 72.4% to 50.7%.

Regarding the metric of total traversal duration, which denotes the time taken for the robot to cover all fruits within the designated partition, this measure also provides insight into the operational effectiveness of the dual-arm system. Evaluation of this parameter indicates that, under the same harvesting conditions, the time expenditure for fruit traversal is invariably reduced when the dynamic partition strategy is applied. Specifically, the minimal total traversal time recorded with dynamic planning is 17.8 s, and the maximal is 22.3 s. In comparison, the shortest duration observed with equal partition planning is 22.8 s, and the longest is 26.2 s.

From the standpoint of the combined path length traversed by both arms, examination reveals that, for the same apple arrangement, the overall path length associated with the equal partition technique is consistently less than that resulting from dynamic partition planning. Moreover, upon assessing the individual path lengths of the left and right manipulators within identical experimental trials, the greatest disparity observed with the dynamic method is 541 mm, and the smallest is 267 mm. Conversely, the maximum discrepancy identified with the equal partition approach is 2294 mm, and the minimum is 1206 mm. Consequently, the variance in path length between the two arms is consistently smaller under dynamic partition planning. Furthermore, the divergence in fruit count allocation between the left and right partitions using the equal method ranges from 2 to 4, while for the dynamic method, it is confined between 0 and 1. This suggests a more equitable distribution of fruit targets is achieved through dynamic partitioning. It should be noted, however, that for the same scenario, the aggregate traversal path length for both arms remains longer with dynamic planning. Across comparable trials, the maximum observed difference in total path length between the two methods is 359 mm, and the minimum is 46 mm.

A holistic assessment indicates that the principal benefit of the ’low-energy consumption-based equal partition planning method for dual-arm harvesting‘ is its energy-efficient characteristic. That is, within the same context, the cumulative distance traveled by the left and right manipulators to accomplish the fruit traversal task is minimized, leading to a reduction in the total kinematic energy expenditure of the robotic system. As the partition boundary in the dynamic method is established according to the spatial configuration of apples in the immediate environment, the harvesting division might deviate from the equidistant line towards either side. This outcome leads to a non-minimal total travel distance for the dual manipulators, thereby augmenting the system’s motion-related energy consumption to a certain degree.

Conversely, the ’pose-constrained dynamic partition planning method for dual-arm harvesting‘ operates on the principle of ensuring that the travel distances for the left and right partitions are as equivalent as possible during task allocation for fruits within the harvestable area. Thus, from an efficiency standpoint, this technique is superior to the low-energy consumption-based equal partition method, guaranteeing that all target fruits are traversed in the minimal achievable time. By maximizing synchronous and parallel operation of the dual arms, the dynamic partition method ensures the highest possible rate of concurrent operation, which in turn enhances the overall harvesting throughput of the robotic system.

For identical orchard configurations, the adaptive zoning methodology yields superior bimanual concurrency ratios relative to static bisection approaches. This metric functions as an efficiency indicator for coordinated manipulator operations, wherein heightened parallelism correlates with elevated aggregate harvesting throughput and increased equipment utilization rates.

Total traversal duration quantifies temporal expenditure for completing zoned harvesting assignments, serving as a complementary efficiency measure. Under equivalent conditions, reduced temporal requirements are observed when employing adaptive zoning compared to static partitioning.

Regarding cumulative traversal distances, path length differentials between manipulators are minimized through adaptive zoning implementation. Furthermore, enhanced distributional equilibrium of targets between operational sectors is achieved. However, aggregate traversal distances for bimanual systems are extended under adaptive zoning relative to static partitioning. This trade-off stems from maximized operational synchronization being prioritized, ensuring peak concurrency proportions and consequent harvesting efficiency improvements.

Comprehensive analysis reveals that demarcation boundaries within the adaptive zoning methodology are established according to spatial target distributions in operational environments. Consequently, harvesting boundaries frequently exhibit lateral extension beyond the bisection axis. Although aggregate traversal duration for bimanual apple harvesting tasks is not minimized—resulting in elevated kinetic expenditure—adherence is maintained to the principle of maximizing inter-zone separation when allocating targets. This approach ensures temporal minimization for completing harvesting assignments within operational envelopes. Furthermore, concurrent manipulator utilization is maximized through this methodology, guaranteeing peak bimanual coordination ratios that enhance aggregate harvesting throughput.

## 4. Conclusions

To facilitate bimanual apple harvesting and enhance robotic operational efficiency, research was undertaken on adaptive zoning and intelligent sequencing methodologies:

(1) A pose-constrained bimanual harvesting zone methodology was introduced, incorporating a U-tube optimization algorithm for target allocation within partially overlapping harvesting regions. Experimental data indicate that for this approach, peak parallel operation ratios reached 99% across four simulation trials, with a minimum ratio of 82.1%. Aggregate traversal distances spanned from 9237 mm to 11,904 mm.

(2) Leveraging horticultural expertise, a sequencing methodology for dynamically zoned bimanual harvesting was developed. Verification was conducted through V-REP simulation environments, where collision-free operation was confirmed during coordinated manipulator movements, validating the safety and reliability of the proposed sequencing approach.

(3) Field validation within Baishui County’s experimental orchard demonstrated that adaptive zoning achieved bimanual concurrency ratios between 85.7% and 93.3%. Temporal requirements spanned 17.8 to 22.3 s, while differential path lengths between manipulators varied from 267 mm (minimal) to 541 mm (maximal).

## 5. Future Work

While the study successfully demonstrated high-efficiency, collision-free dual-arm operation, there are still several limitations: Fruit Homogeneity—the U-tube optimization currently prioritizes spatial efficiency without penalizing size variability or tree crown-access complexity, potentially affecting success rates. Energy Optimization—trajectory planning minimizes path length but does not yet optimize for energy consumption or joint wear.

The future focus will be on introducing and analyzing additional factors (such as maturity differences and picking difficulty) into the dual-arm picking–sorting process, establishing a more comprehensive decision-making model, and achieving more efficient and scientific planning. The objective function prioritized critical operational metrics, minimizing total path length and maximizing parallelization while ensuring safety, which is a simplification to establish baseline viability. Future work will also integrate dynamic penalties of the U-shaped tube optimal allocation algorithm (e.g., energy cost, fruit size variance) as we advance toward unstructured orchard environments, building upon this foundational proof of concept. Further, we will promote the deep integration of picking robots with orchard information management systems, achieve real-time data synchronization and analysis, and empower the precision management practice for orchards.

Building upon the validated efficacy of the U-tube-based dynamic zoning and sequencing methodology for dual-manipulator apple harvesting demonstrated in this research, several promising avenues for future development emerge, poised to significantly enhance the robustness, efficiency, scalability, and practical viability of intelligent orchard robotic systems. A primary focus will be on expanding environmental adaptability and robustness. Future work should rigorously test the system under a wider spectrum of challenging orchard conditions, including dense foliage, varying lighting conditions (dawn, dusk, high glare), diverse fruit occlusion scenarios (clusters, leaves), and adverse weather influences like wind. Integrating more advanced, real-time 3D perception systems, potentially leveraging multi-sensor fusion (e.g., combining RGB-D cameras with LiDAR or thermal imaging), will be crucial for reliable fruit detection, localization, and stem identification under these complex, non-ideal circumstances. Furthermore, enhancing manipulator dexterity and end-effector intelligence represents a critical frontier. Developing next-generation end-effectors capable of gentle yet secure grasping of fruits with significant size, shape, and ripeness variation, incorporating tactile feedback for grip force control, and reliably executing precise stem cutting or detachment without damaging the fruit is essential for reducing losses and increasing harvest quality. Research into adaptive control strategies that allow the manipulators to react to minor fruit movement (e.g., caused by wind or contact) during the approach and grasp phases would significantly improve success rates.

Concurrently, optimizing system-level intelligence and coordination beyond the current framework is vital. Exploring more sophisticated multi-agent task allocation and scheduling algorithms, potentially incorporating machine learning to predict harvest times or fruit accessibility based on real-time sensor data and historical performance, could further minimize total cycle time and maximize parallel operation efficiency. Extending the concept to collaborative multi-robot systems involving more than two manipulators, either on a single platform or across coordinated mobile units, necessitates research into fleet coordination, collision avoidance in shared 3D space, and dynamic workload balancing to unlock unprecedented throughput potential for large-scale orchards. Investigating real-time adaptability and learning is another key direction. Implementing systems that can learn from successful and failed harvest attempts to continuously refine motion planning parameters, gripping strategies, or even the U-tube zoning rules for specific orchard blocks or tree architectures would lead to progressively better performance. Finally, improving operational practicality and integration demands attention. Future efforts should focus on enhancing the robot’s mobility and navigation capabilities within complex orchard terrains, developing efficient and autonomous recharging/power management solutions, simplifying system calibration and maintenance, and rigorously evaluating the long-term economic viability, including durability testing and total cost of ownership analysis compared to manual labor and alternative technologies. Addressing these multifaceted challenges will be instrumental in transitioning this promising dual-arm robotic harvesting technology from successful prototypes to reliable, high-performance solutions ready for widespread adoption in modern, intelligent orchards.

## Figures and Tables

**Table 1:** Analysis of dangerous/safe picking posture constraints for the left and right arms.

	Manipulator Joint	Joint 1 Angle	Joint 3 Angle
Manipulator Attitude			
Left mechanical arm	Dangerous picking posture	(−90°, 90°)	>0°
	Safe picking posture constraint	(−90°, 90°)	<0°
Right mechanical arm	Dangerous picking posture	(−90°, 90°)	<0°
	Safe picking posture constraint	(−90°, 90°)	>0°

**Table 2:** Experimental results of the dynamic zoning planning method for double-arm picking.

Test Group	Sum Distance/Number of Fruits Distributed		Total Length of Dual-Arm Operation Path (mm)	Left-Arm Operation Path Length (mm)	Right-Arm Operation Path Length (mm)	Maximum Proportion of Parallel Tasks for Dual Arms (%)	Maximum Single-Arm Operation Length (mm)
1	left zone ≥ right zone	left zone: 9 right zone: 9 semi-overlapping space: 2	10,703	5080	5623	90.3	5623
	left zone < right zone	left zone: 7 right zone: 8 semi-overlapping space: 2	9237	4404	4833	91.1	4833
2	left zone ≥ right zone	left zone: 8 right zone: 9 semi-overlapping space: 1	9894	4856	5038	96.4	5038
	left zone < right zone	left zone: 10 right zone: 9 semi-overlapping space: 1	11,229	5641	5588	99	5641
3	left zone ≥ right zone	left zone: 9 right zone: 11 semi-overlapping space: 1	11,904	5367	6537	82.1	6537
	left zone < right zone	left zone: 8 right zone: 8 semi-overlapping space: 2	9611	4599	5012	91.8	5012
4	left zone ≥ right zone	left zone: 10 right zone: 10 semi-overlapping space: 2	11,805	5706	6099	93.6	6099
	left zone < right zone	left zone: 9 right zone: 9 semi-overlapping space: 1	11,263	5602	5661	99	5661

**Table 3:** Orchard experiment of robot operation based on dynamic picking zoning method.

Test Group	Distribution of Fruit Quantity in Left and Right Zones	Total Length of Dual-Arm Traversal Path (mm)	Left-Arm Traversal Path Length (mm)	Right-Arm Traversal Path Length (mm)	Maximum Proportion of Parallel Tasks for Both Arms (%)	Total Traversal Time of Robot (s)
1	left zone: 7 right zone: 7	7647	3957	3690	93.3	18.6
2	left zone: 6 right zone: 6	6684	3084	3600	85.7	17.8
3	left zone: 8 right zone: 8	8963	4752	4211	88.6	22.3
4	left zone: 7 right zone: 8	7185	3428	3757	91.2	21.0

## Data Availability

The original contributions presented in the study are included in the article; further inquiries can be directed to the corresponding author.
